# Prognostic Significance of Cyclin D1 Expression in Small Intestinal Adenocarcinoma

**DOI:** 10.3390/cancers15205032

**Published:** 2023-10-18

**Authors:** Sun-Young Jun, Seung-Mo Hong, Kee-Taek Jang

**Affiliations:** 1 Department of Pathology, Incheon St. Mary’s Hospital, College of Medicine, The Catholic University of Korea, Seoul 21431, Republic of Korea; 2 Department of Pathology, Asan Medical Center, University of Ulsan College of Medicine, Seoul 05505, Republic of Korea; smhong28@gmail.com; 3 Department of Pathology, Samsung Medical Center, Sungkyunkwan University School of Medicine, Seoul 06351, Republic of Korea; kt12.jang@samsung.com

**Keywords:** small intestine, adenocarcinoma, cyclin D1, immunohistochemistry, survival, prognosis

## Abstract

**Simple Summary:**

Small intestinal adenocarcinoma (SIAC) is a rare tumor, with a rising incidence in recent decades. Although the National Comprehensive Cancer Network (NCCN) established the treatment guidelines for SIAC in 2020, there continues to be urgency to search for reliable prognostic factors and therapeutic regimens. Cyclin D1, a critical cyclin-dependent kinase (CDK) 4/6-dependent regulator of G1/S transition, has attracted much interest as a therapeutic target. The cyclin D1 expression in SIACs has not yet been comprehensively studied, owing to the rarity of this tumor. We investigated the clinicopathological and prognostic significance of the cyclin D1 expression in 232 primary SIACs through a multi-institutional study. Cyclin D1 was commonly overexpressed in SIACs, and a high expression of cyclin D1 was identified as a favorable prognostic indicator in SIAC patients. These findings in SIACs may be important to further the understanding of the mechanism of cyclin D1 in carcinogenesis and for applying appropriate patient therapies.

**Abstract:**

Cyclin D1, a critical cyclin-dependent kinase (CDK) 4/6-dependent regulator of G1/S transition, has attracted much interest as a therapeutic target. The cyclin D1 expression in small intestinal adenocarcinomas (SIACs) has not yet been comprehensively studied, owing to the rarity of this tumor. We investigated the clinicopathological and prognostic significance of the cyclin D1 expression in 232 surgically resected primary SIACs through a multi-institutional study. A high expression of cyclin D1 (cyclin D1^High^) was detected in 145 SIAC cases (63%), which was significantly higher than that in normal small intestinal mucosa (11%). Cyclin D1^High^ was more commonly found in SIACs with a lower T-category and disease stage and *KRAS* mutation and predicted better patient survival. Multivariate analysis revealed that cyclin D1^High^, the absence of retroperitoneal seeding and lymphovascular invasion, and the lower N-category were identified as independent prognostic indicators for patients with SIACs. Specifically, cyclin D1^High^ affected patient survival in the lower stage group (stages I and II). In conclusion, cyclin D1 was commonly overexpressed in SIACs, and cyclin D1^High^ acted as a favorable prognostic indicator in patients with SIACs. These findings in SIACs may, thus, be important to further comprehend the mechanism of cyclin D1 in carcinogenesis and to strategize appropriate patient therapies.

## 1. Introduction

Uncontrolled cell proliferation via dysregulation of the cell cycle is a hallmark of cancer [1]. Thus, the cell cycle regulation mechanism in cancer is a topic of enormous interest. Cyclin D1, which is encoded by *CCND1*, is a critical regulator of the G1/S transition and promotes cancer progression in a complex with cyclin-dependent kinase (CDK) 4/6 [2]. Cyclin D1 is overexpressed in various types of cancers; however, findings on its prognostic impact remain inconclusive [3]. A recent meta-analysis of 108 original studies revealed that cyclin D1 had varying effects on prognosis, depending on the cancer site [3]. The cyclin D1 overexpression was related to worse survival in patients with head and neck cancers, but not in patients with gastrointestinal (GI) tract, breast, bladder, or lung cancers [3]. Furthermore, an opposite prognostic effect of cyclin D1 was identified between estrogen receptor (ER)-positive and ER-negative breast cancers [4] and between superficial and muscle-invasive bladder cancers [5]. As expected, the cyclin D1 overexpression was not always accompanied by *CCND1* amplification, which implied additional mechanisms of cyclin D1 overexpression beyond *CCND1* amplification, such as the activation of mitogenic signaling pathways [3,6]. Ishii et al. proposed that cyclin D1 may improve the outcomes by bypassing its conservative oncogene function through the direct inhibition of the oncogenic signal transducer and activator of transcription 3 (STAT3) [7]. Therefore, the contribution of cyclin D1 to carcinogenesis remains unclear.

Small intestinal adenocarcinoma (SIAC) is extremely rare. In the United States, there will be 12,070 new cases of SIAC in 2023, accounting for 0.6% of all new cancer cases [8]. In Korea, the incidence of new cases of SIAC is lower than that in the United States, with an estimated 958 new cases occurring in 2020, which accounted for 0.3% of all new cancer cases [9]. In localized SIACs, surgical resection is the best approach to improve survival [10]. Systemic treatment for SIAC is mainly extrapolated from the management of colorectal carcinoma (CRC) due to the embryonic developmental similarity [11]. Previous studies on SIACs have compared them to CRCs [12] and a few prognostic factors of SIACs have been identified, which include tumor location, microsatellite instability (MSI), and *KRAS* mutations [13,14,15]. Based on the limited data available from retrospective studies on SIAC and extrapolation from studies on CRC, the National Comprehensive Cancer Network (NCCN) was the first to establish a standard treatment guideline for SIAC in 2020 [16]. The NCCN recommended 5-fluorouracil (5-FU) and leucovorin (LV), 5-FU/LV plus oxaliplatin (FOLFOX), capecitabine plus oxaliplatin (CAPEOX), or capecitabine as adjuvant chemotherapy. However, the efficacy of these combinations in SIAC is variable [16]. Therefore, there continues to be a pressing need for reliable prognostic factors and therapeutic regimens for patients with SIAC.

Previous studies on cyclin D1 in SIAC are extremely rare, although numerous studies have been reported on cyclin D1 expression in CRC [3,17,18]. Furthermore, the CDK4/6 inhibitors—ribociclib, palbociclib, and abemaciclib—have been evaluated in CRC, and their therapeutic effect has been amplified in combination with other drugs [19]. In experimental studies, *KRAS*-mutated CRCs and *RAS*- and *BRAF*-mutant CRCs were found to be sensitive to a combination of palbociclib with MEK inhibitor and a combination of abemaciclib and RAF inhibitor, respectively [20,21]. These promising results from CDK4/6 inhibitors in experimental models were followed by studies for their efficacy in clinical trials. Currently, the combination of abemaciclib with ERK1/2 inhibitor and cetuximab and the combination of CDK4/6 inhibitor with checkpoint inhibitors such as anti-programmed cell death 1 (PD1) therapy is being clinically tested in patients with advanced CRC [19]. Although several studies on cyclin D1 expression have been actively conducted before, considering the application of CDK4/6 inhibitors in CRC, cyclin D1-related studies in SIAC remain limited and rudimentary, owing to the rarity of the disease. To the best of our knowledge, there was only one SIAC study on cyclin D1, which was performed in a small cohort of 24 SIAC cases [22].

Thus, this study aimed to investigate the clinicopathological and prognostic significance of cyclin D1 in SIACs collected through a multi-institutional study. In addition, we compared the cyclin D1 expression in SIACs with that in normal small intestine and evaluated the association of cyclin D1 with *KRAS* and *BRAF* mutations.

## 2. Materials and Methods

### 2.1. Study Population

A total of 236 surgically resected primary SIACs were collected from the surgical pathology archives of 23 South Korean institutions by the Korean Small Intestinal Cancer Study Group, as previously reported [23]. Carcinomas originating in the duodenum, jejunum, and ileum were included, but tumors extending into the small intestines from the surrounding GI tract organs were excluded. Of the 236 SIAC cases, four cases without available tissue blocks were excluded; hence, the study finally included 232 SIACs. The clinicopathological findings of the patients were updated, including the most recent follow-up data, survival status, and TNM staging in accordance with the 8th American Joint Committee on Cancer (AJCC) staging system [24].

### 2.2. Immunohistochemical Analysis

For immunohistochemistry (IHC), tissue microarrays (TMAs) were constructed containing three cores of each tumor and one core of possible paired normal mucosa [18]. The TMA sections were immunostained using an anti-cyclin D1 antibody (clone SP4; Cell Marque, Darmstadt, Germany) and the Ventana BenchMark XT immunostainer (Ventana Medical System, Tucson, AZ, USA). The nuclear expression of cyclin D1 was semiquantitatively determined by intensity and percentage, as described previously [18]. The staining intensity was graded as 0 (no staining), 1 (weak staining), 2 (moderate staining), and 3 (strong staining). The percentage of staining was graded on a scale of 0–10, at 10% increments of 0 (no staining), 1 (1–10%), 2 (11–20%), and 3 (21–30%), up to 10 (91–100%). The staining intensity grade (0–3) was multiplied by the percentage grade of the stained cells (0–10) to yield an immunostaining score ranging from 0 to 30. The average score for each case was calculated and selected as the final overall score.

### 2.3. Molecular Analysis

We obtained information about mutations in codons 12 and 13 of *KRAS* exon 1 and codon 600 of *BRAF* exon 15 from elsewhere [15]. Genomic DNA was extracted with the QIAmp DNA Mini Kit (Qiagen, Valencia, CA, USA), and *KRAS* and *BRAF* mutations were analyzed by cycle sequencing [15].

### 2.4. Statistical Analysis

Statistical analyses were performed using Statistics for Windows (version 28.0; IBM, Armonk, NY, USA). The association of the cyclin D1 expression with clinicopathological factors was evaluated using the unpaired Student’s *t*-test for continuous data and the χ^2^ and/or Fisher’s exact test for categorical data. The survival curves were plotted using the Kaplan–Meier method, and the associations between the overall survival (OS) rates and various clinicopathological factors were assessed using the log-rank test. The Cox proportional hazards model was used to calculate the significance of any prognostic factors. Receiver operating characteristic (ROC) curves were generated to evaluate the predictive power of the cyclin D1 expression for accurately classifying SIAC cases related to OS. *p* < 0.05 were considered to indicate statistical significance.

## 3. Results

### 3.1. Clinicopathological Characteristics

The baseline clinicopathological characteristics are shown in Table 1. The mean patient age was 59.9 ± 12.8 years (range: 23–84 years), and the male-to-female ratio was 1.8. The tumors, which ranged in size from 0.5 to 16.0 cm (mean size: 4.3 ± 2.5 cm), were located in the duodenum (140 cases, 60.3%), jejunum (58, 25.0%), and ileum (34, 14.7%). An infiltrative growth pattern was observed in 74.5% of the tumors (167/224), followed by polypoid (40/224, 17.9%) and nodular (17/224, 7.6%) patterns. The majority of the tumors were tubular adenocarcinoma (205/232, 88.4%) and of low grade (184/232, 79.3%). Nontubular carcinomas were also detected, which included 12 (5.2%) mucinous carcinomas, six (2.6%) medullar carcinomas, five (2.1%) undifferentiated carcinomas, and four (1.7%) signet ring cell carcinomas. Pancreatic invasion was observed in 87 cases (37.5%). Six cases (2.6%) of other loop invasions and 16 cases (6.9%) of retroperitoneal invasion were seen. Lymphovascular and perineural invasions were evaluated in 231 cases and, respectively, observed in 113 (48.9%) and 76 (32.9%) cases. Resection margins with cancer involvement were noted in nine cases (9/218, 4.1%). According to the AJCC staging scheme, four cases (1.7%) were categorized as Tis, 13 cases (5.6%) as T1, 14 cases (6.1%) as T2, 68 cases (29.3%) as T3, and 133 cases (57.3%) as T4 tumors. The nodal status was investigated in 215 cases. Of these, nodal metastases were detected in 49.3% (106/215) of the tumors, including 54 cases (25.1%) of N1 and 52 cases (24.2%) of N2. Consequently, the tumors were grouped into four cases (4/215, 1.9%) of stage 0, 22 cases (10.2%) of stage I, 83 cases (38.6%) of stage II, and 106 cases (49.3%) of stage III. No case of stage IV was noted in this study. *KRAS* was mutated in 32.3% (60/186) of the tumors, while *BRAF* mutations were observed in 1.1% (2/176) of the tumors. Chemotherapy and radiotherapy were performed in 36.1% (82/227) and 11.5% (26/226) of the patients, respectively. The follow-up period after surgical resection was 1.1–168.4 months (mean: 41.5 ± 40.9 months).

### 3.2. Cyclin D1 Expression

**Figure 1:**
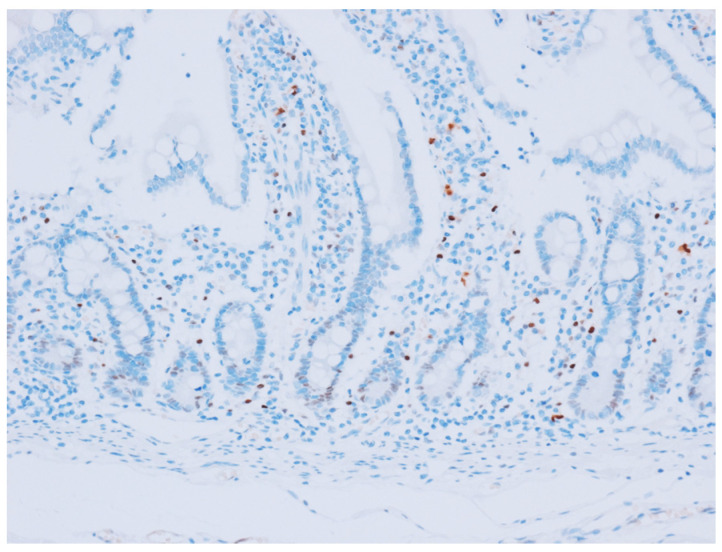
Cyclin D1 expression in normal mucosa of the small intestine. The cyclin D1 was expressed in the transitional zone of the crypt at the lower portion of the gland of normal small intestinal mucosa (original magnification ×200).

Cyclin D1 immunostaining was interpretable in 189 cases of normal mucosa of the small intestine. Of these, 79 cases (41.8%) expressed cyclin D1 in the transitional zone of the crypt at the lower portion of the gland (Figure 1), which consisted of 47 cases (47/79, 59.5%) with weak staining intensity, 31 (39.2%) with moderate intensity, and 1 (1.3%) with strong intensity. The cyclin D1 staining score in the normal epithelia ranged from 1 to 12, which included 35 cases (35/79, 44.3%) with a score of 1, 18 cases (22.8%) with a score of 2, 11 cases (13.9%) with a score of 4, five cases (6.3%) each with a score of 3 or 8, three cases (3.8%) with a score of 6, and one case (1.3%) each with a score of 5 or 12.

**Figure 2:**
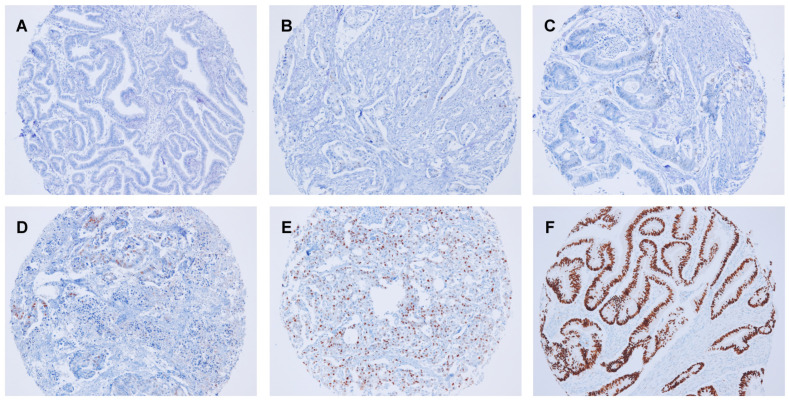
Representative images of cyclin D1 expression in SIAC. Cyclin D1^Low^ showed (**A**) a score of 0, (**B**) a score of 1, and (**C**) a score of 2. Cyclin D1^High^ had (**D**) a score of 14, (**E**) a score of 18, and (**F**) a score of 30 (original magnification ×100).

Of the 232 SIAC cases, cyclin D1 was expressed in 220 (94.8%). The cyclin D1 staining scores in SIACs ranged from 0.3 to 30 in the following order: 0 < score ≤ 5 in 95 cases (95/220, 43.2%); 5 < score ≤ 10 in 32 cases (32/220, 14.6%); 10 < score ≤ 15 in 33 cases (33/220, 15.0%); 15 < score ≤ 20 in 28 cases (28/220, 12.7%); 20 < score ≤ 25 in 19 cases (19/220, 8.6%); 25 < score ≤ 30 in 13 cases (13/220, 5.9%). The cyclin D1 staining scores of SIACs were significantly higher than those of the normal mucosa of the small intestine (mean: 9.5 ± 8.4 vs. 1.0 ± 1.9; *p* < 0.001). Most (210/220, 95.5%) SIACs had a score of ≥1. Based on the ROC curve analysis, a high expression of cyclin D1 (cyclin D1^High^) was defined as a staining score of >3.5. Cyclin D1^High^ was detected in 145 SIAC cases (62.5%) (Figure 2A–F), which was more often than reported in normal small intestinal mucosa (21/189, 11.1%; *p* < 0.001). The relationship between cyclin D1^High^ and the clinicopathological factors of SIAC patients is described in Table 1. Cyclin D1^High^ was more commonly found in tumors with a lower T category (*p* = 0.046) and disease stage (*p* = 0.003) and *KRAS* mutations (*p* = 0.026).

### 3.3. Survival Analysis

**Figure 3:**
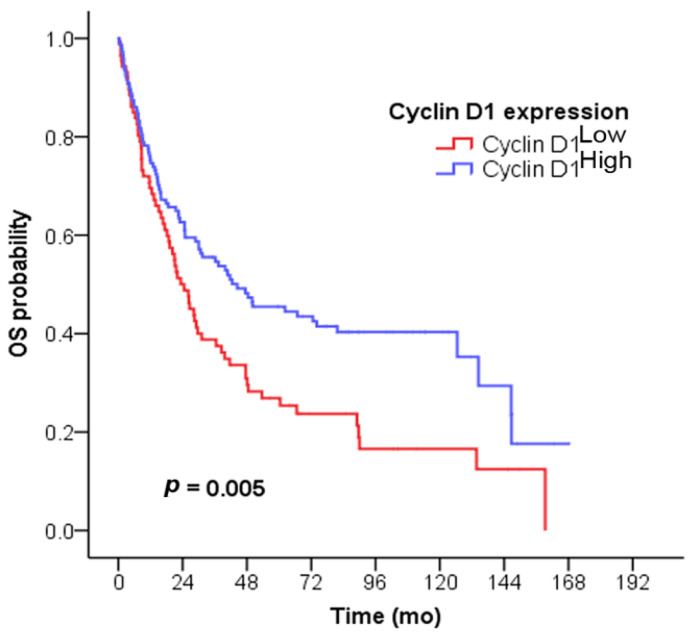
Univariate survival analysis of cyclin D1^High^ in SIAC patients. The patients with cyclin D1^High^ had significantly longer survival times than those with cyclin D1^Low^ (*p* = 0.005).

The survival analysis results are summarized in Table 2. By univariate survival analysis, SIAC patients with cyclin D1^High^ showed significantly longer survival times than those with cyclin D1^Low^ (median, 44.4 months vs. 24.5 months, *p* = 0.005; Figure 3). In addition, proximal tumor location (*p* = 0.007), the absence of other loop invasion (*p* = 0.044) or retroperitoneal seeding (*p* < 0.001), no lymphovascular (*p* < 0.001) or perineural invasion (*p* = 0.004), no radiotherapy (*p* = 0.005), no nodal metastasis (*p* < 0.001), and lower T and N categories and disease stage (all *p* < 0.001) were all related to better OS. In the multivariate analysis, cyclin D1^High^ (*p* = 0.031), the absence of retroperitoneal seeding (*p* = 0.007) or lymphovascular invasion (*p* = 0.010), and lower N category (*p* = 0.001) were identified as favorable prognostic indicators for patients with SIACs.

**Figure 4:**
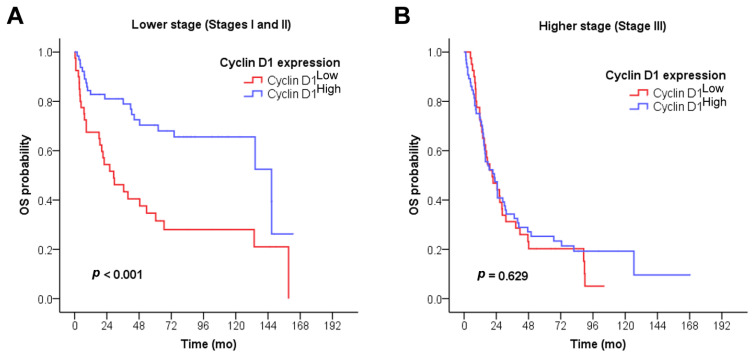
Prognostic impact of cyclin D1 expression in SIAC depending on tumor stage. (**A**) Cyclin D1^High^ significantly predicted the longer OS of patients in the lower stage group (stages I and II, *n* = 105; *p* < 0.001), while (**B**) no significant difference in survival was identified between the cyclin D1^High^ and cyclin D1^Low^ groups in the higher stage group (stage III, *n* = 106; *p* = 0.629).

Further, the prognostic impact of cyclin D1 expression was investigated with respect to tumor stage (Figure 4A,B). Cyclin D1^High^ significantly predicted the better OS of SIAC patients in the lower stage group. In the lower stage group (stages I and II, *n* = 105), SIAC patients with cyclin D1^High^ had significantly longer survival times (median, 146.6 months) than those with cyclin D1^Low^ (29.1 months; *p* < 0.001; Figure 4A). In the higher stage group (stage III, *n* = 106), no significant difference in OS was identified between the cyclin D1^High^ and cyclin D1^Low^ groups (22.6 months vs. 21.0 months, *p* = 0.629; Figure 4B).

## 4. Discussion

Despite our meticulous search for reports of cyclin D1 in SIAC through PubMed, Embase, and Google Scholar, with further searches via manual cross-referencing, we found only one study by Arber et al. conducted in a small cohort of 24 cases [22]. In the SIAC study by Arber and colleagues, they used a non-SP4 clone and found that cyclin D1^High^ was associated with a decrease in 3-year survival rates of patients [22]. Meanwhile, in the present study, we evaluated cyclin D1 expression using SP4 in 232 SIACs and found that cyclin D1^High^ predicted better survival outcomes. As mentioned earlier, SIAC has been studied comparatively with CRC because of its embryonic developmental similarity and anatomical proximity. Given the rarity of SIAC studies on cyclin D1, we alternatively analyzed the present study in comparison with previous CRC studies on cyclin D1 (Supplementary Table S1). Conflicting results have been reported regarding the prognostic effect of cyclin D1 expression in CRCs [18]. The heterogeneity in these results may have stemmed from differences in sample sizes, tissue section type, the clone used, and cut-off values for cyclin D1 expression [18].

To analyze the effect of the clones used, we investigated reports of cyclin D1 IHC in malignancies [25,26,27]. Cyclin D1 IHC has been a key tool in distinguishing mantle cell lymphoma from other small B cell lymphomas but suffers from technical difficulties and ambiguous staining results [25]. Cheuk et al. first demonstrated the superior performance of the newly available rabbit monoclonal antibody SP4 for cyclin D1 IHC in 2004 [25]. In subsequent comparative studies with various anti-cyclin D1 antibodies, SP4 produced the strongest staining with a high sensitivity of 95%, enabling the optimal detection of cyclin D1 expression [26,27]. In CRC, five studies on the prognostic effect of cyclin D1 expression using SP4 were found (Supplementary Table S1) [18,28,29,30,31]. All five studies were conducted with TMAs. Their mean frequency of cyclin D1^High^ was 65.0% (range, 54.0%–78.6%), similar to the 62.5% found in the present study of SIACs. Three of five CRC studies revealed a favorable prognostic role of cyclin D1, and all of these were performed in relatively large CRC cohorts containing >200 cases [18,28,29]. In contrast, the other two studies on <200 CRC cases did not find any prognostic significance of cyclin D1 [30,31]. When searching studies of cyclin D1 expression using non-SP4 clones in CRCs, we found 18 studies (Supplementary Table S1) [32,33,34,35,36,37,38,39,40,41,42,43,44,45,46,47,48,49]. Most studies (15/18, 83.3%) performed cyclin D1 immunostaining on conventionally sectioned slides [33,34,35,36,37,38,39,40,41,42,43,45,47,48,49]. Nine of 18 studies precisely defined the clones [32,33,34,35,36,37,38,39,40], and two of them on >200 cases consistently showed an association between cyclin D1 and better prognosis [32,33]. To obtain reliable results for the prognostic significance of cyclin D1, we designed a multi-institutional study and selected SP4 for use. We constructed TMAs for IHC because no heterogeneity of cyclin D1 expression was observed in selected SIAC cases. Expectedly, cyclin D1^High^ effectively predicted better prognoses of patients with SIACs. A limitation of this study is that it was conducted in a Korean population with homogeneous ethnic group and there is a lack of comparable studies on cyclin D1 in SIAC. Nevertheless, we assessed cyclin D1 expression in a relatively large cohort of SIAC patients and identified the associations of cyclin D1^High^ with lower T category and disease stage and better survival outcomes. We also found that cyclin D1^High^ significantly predicted the better OS of SIAC patients in the lower stage group. Further studies with a large number of SIAC cases are needed to solidify the prognostic predictive value of cyclin D1.

Various cutoff points for cyclin D1^High^ have been applied in both CRC and SIAC studies, and cyclin D1^High^ has often been interpreted using predetermined cutoffs, such as 5% or 10% cell positivity without relevant information (Supplementary Table S1) [30,33,34,35,39,40,45,46,47,48,49]. The SIAC study by Arber et al. also defined cyclin D1^High^ when staining was present in at least 10% of the tumor, irrespective of the intensity, without any further explanation [22]. Considering the lack of any standardized criteria for cyclin D1 overexpression, we performed ROC analysis to establish the objectivity and optimal decision threshold, which is very necessary for IHC interpretation [50]. When using a staining score cutoff point of >3.5, the area under the curve was 0.610. A substantial accuracy in predicting OS was detected, with a sensitivity of 45.3% and a specificity of 76.2%. Accumulating results from cyclin D1 IHC studies in various carcinomas based on ROC analysis can help establish clear and consistent criteria for assessing cyclin D1 expression in the future. Interestingly, Arber et al. observed the cyclin D1 expression at the base of the crypt of normal epithelia of the small intestine [22]; however, they did not compare the expression of cyclin D1 between the normal mucosa and SIAC. In this study, we observed that cyclin D1 was identically expressed in the transitional zone of the crypt at the lower portion of the normal gland and identified cyclin D1^High^ in 11% (21/189) of the normal small intestinal mucosa. Both the cyclin D1 composite staining score and the frequency of cyclin D1^High^ were consistently higher in SIACs than in normal mucosa of the small intestine.

The amplification of *CCND1* as an oncogene is known to promote growth and carcinogenesis by regulating cyclin D1 [3]. In contrast, the overexpression of cyclin D1 is not always accompanied by DNA amplification. *CCND1* is amplified in 5–20% of all malignancies, but cyclin D1 is more prevalently overexpressed, with a reported frequency of up to 80% [6,51]. Several unexpected functions of cyclin D1 in addition to the oncogenic effects have been reported [52,53,54]. The upregulation of cyclin D1 in breast cells inhibited growth by a prolonged S-phase via retinoblastoma tumor suppressor protein (pRB) and proliferating cell nuclear antigen (PCNA)-mediated DNA synthesis or repair [52]. In addition, cyclin D1 was involved in programmed cell death [53] and could suppress the proliferation of diploid fibroblasts [54]. Ogino et al. hypothesized that cyclin D1-negative CRCs may bypass cyclin D1 activation and develop more aggressive behavior than cyclin D1-positive tumors by accumulating multiple genetic and epigenetic events during carcinogenesis [29].

Previous in vitro studies have described that mutated *KRAS* upregulated the cyclin D1 expression through pathways involving RAS-MEK-ERK and PI3K signaling cascades [55,56]. An association between cyclin D1 mRNA expression and *KRAS* mutation was also reported [56]. We examined *CCND1* mutations in a part of our cohort by next-generation sequencing (*n* = 97) and identified no *CCND1* mutation. However, we did identify an association between cyclin D1^High^ and *KRAS* mutations in this study. These findings support the presence of additional mechanisms of cyclin D1 overexpression beyond *CCND1* amplification. Dragnev et al. reported that *KRAS*-driven lung cancer, which usually responds poorly to the epidermal growth factor receptor (EGFR) inhibitor erlotinib, was particularly dependent on CDK4 and was sensitive to the cyclin D1-degrading combination of bexarotene and erlotinib [57]. In CRCs, *KRAS*-mutant tumors are particularly sensitive to a combination of mitogen-activated protein kinase (MAPK) and CDK4/6 inhibitors [58]. Therefore, inhibiting the cyclin D1–CDK4/CDK6 pathway may enhance responses to targeted therapy, particularly in *KRAS*-mutated SIACs. Further studies are, however, warranted to elucidate the role of cyclin D1 in the carcinogenesis of SIAC and the interaction of cyclin D1 with other signaling pathways.

A few studies have noted a relationship between the cyclin D1 expression and the response to cancer therapy [55]. In experimental models, the cyclin D1 overexpression induced radio-resistance [59] and resistance to cytotoxic drugs [60], antiestrogens [61], and an EGFR tyrosine kinase inhibitor [62], as well as inhibitors of BRAF and MEK signaling [63]. An association between the cyclin D1 overexpression and therapeutic response to drugs, such as tamoxifen in breast cancer patients [64] and combined chemotherapy of erlotinib and bexarotene in lung cancer patients, was identified in clinical studies [57]. Cyclin D1 may, thus, help control the therapeutic effect in patients with SIAC.

## 5. Conclusions

Cyclin D1 was commonly overexpressed in SIACs, and cyclin D1^High^ was a favorable prognostic indicator in patients with SIACs. These findings about SIACs may be important to further understand the mechanism of cyclin D1 in carcinogenesis and to strategize appropriate patient therapies.

## Figures and Tables

**Table 1:** Association between cyclin D1 expression and clinicopathological factors in SIAC.

Characteristics, *n* (%)		Total	Cyclin D1^Low^	Cyclin D1^High^	*p*
No. of patients			87 (37.5)	145 (62.5)	
Age (y)	≤50	52 (22.4)	25 (48.1)	27 (51.9)	0.074
	>50	180 (77.6)	62 (34.4)	118 (65.6)	
Sex	Male	148 (63.8)	58 (39.2)	90 (60.8)	0.481
	Female	84 (36.2)	29 (34.5)	55 (65.5)	
Tumor size (cm, mean ± SD)			4.3 ± 2.4	4.4 ± 2.6	0.200
Growth pattern (*n* = 224) ^a^	Polypoid	40 (17.9)	11 (27.5)	29 (72.5)	0.164
	Nodular	17 (7.6)	9 (52.9)	8 (47.1)	
	Infiltrative	167 (74.5)	66 (39.5)	101 (60.5)	
Tumor location	Duodenum	140 (60.3)	55 (39.3)	85 (60.7)	0.289
	Jejunum	58 (25.0)	17 (29.3)	41 (70.7)	
	Ileum	34 (14.7)	15 (44.1)	19 (55.9)	
Histologic type	Tubular	205 (88.4)	74 (36.1)	131 (63.9)	0.489
	Mucinous	12 (5.2)	6 (50.0)	6 (50.0)	
	Signet ring cell	4 (1.7)	3 (75.0)	1 (25.0)	
	Medullary	6 (2.6)	2 (33.3)	4 (66.7)	
	Undifferentiated	5 (2.1)	2 (40.0)	3 (60.0)	
Differentiation	Low grade	184 (79.3)	64 (34.8)	120 (65.2)	0.094
	High grade	48 (20.7)	23 (47.9)	25 (52.1)	
Pancreatic invasion	Present	87 (37.5)	37 (42.5)	50 (57.5)	0.220
Other loop invasion	Present	6 (2.6)	2 (33.3)	4 (66.7)	1.000
Retroperitoneal seeding	Present	16 (6.9)	9 (56.2)	7 (43.8)	0.108
Lymphovascular invasion (*n* = 231) ^a^	Absent	118 (51.1)	38 (32.2)	80 (67.8)	0.106
	Present	113 (48.9)	48 (42.5)	65 (57.5)	
Perineural invasion (*n* = 231) ^a^	Absent	155 (67.1)	53 (34.2)	102 (65.8)	0.173
	Present	76 (32.9)	33 (43.4)	43 (56.6)	
Margin status (*n* = 218) ^a^	No involvement	209 (95.9)	78 (37.3)	131 (62.7)	0.491
	Involved by cancer	9 (4.1)	2 (22.2)	7 (77.8)	
Chemotherapy (*n* = 227) ^a^	Absent	145 (63.9)	49 (33.8)	96 (66.2)	0.183
	Present	82 (36.1)	35 (42.7)	47 (57.3)	
Radiotherapy (*n* = 226) ^a^	Absent	200 (88.5)	71 (35.5)	129 (64.5)	0.150
	Present	26 (11.5)	13 (50.0)	13 (50.0)	
Nodal metastasis (*n* = 215) ^a^	Absent	109 (50.7)	40 (36.7)	69 (53.3)	0.764
	Present	106 (49.3)	41 (38.7)	65 (61.3)	
T category	Tis	4 (1.7)	0	4 (100)	0.046 ^b^
	T1	13 (5.6)	1 (7.7)	12 (92.3)	
	T2	14 (6.1)	4 (28.6)	10 (71.4)	
	T3	68 (29.3)	25 (36.8)	43 (63.2)	
	T4	133 (57.3)	57 (42.9)	76 (57.1)	
N category (*n* = 215) ^a^	N0	109 (50.7)	40 (36.7)	69 (63.3)	0.865
	N1	54 (25.1)	22 (40.7)	32 (59.3)	
	N2	52 (24.2)	19 (36.5)	33 (63.5)	
Stage grouping (*n* = 215) ^a^	Stage 0	4 (1.9)	0	4 (100)	0.003 ^b^
	Stage I	22 (10.2)	2 (9.1)	20 (90.9)	
	Stage II	83 (38.6)	38 (45.8)	45 (54.2)	
	Stage III	106 (49.3)	41 (38.7)	65 (61.3)	
*KRAS* (*n* = 186) ^a^	Absent	126 (67.7)	55 (43.7)	71 (56.3)	0.026 ^b^
	Present	60 (32.3)	16 (26.7)	44 (73.3)	
*BRAF* (*n* = 176) ^a^	Absent	174 (98.9)	66 (37.9)	108 (62.1)	
	Present	2 (1.1)	1 (50.0)	1 (50.0)	

Low expression of cyclin D1 (cyclin D1^Low^); high expression of cyclin D1 (cyclin D1^High^); standard deviation (SD); ^a^ only calculated using cases with available information; ^b^ significant at *p* < 0.05.

**Table 2:** Association between clinicopathological factors and OS in SIAC.

Characteristics		Univariate		Multivariate	
		Median (mo)	*p*	HR (95% CI)	*p*
Cyclin D1 expression	Cyclin D1^Low^	24.5	0.005 ^a^	0.68 (0.47–0.96)	0.031 ^a^
	Cyclin D1^High^	44.4			
Age (y)	≤50	39.9	0.143		
	>50	30.0			
Sex	Male	30.0	0.873		
	Female	31.4			
Tumor size (cm)		1.00 (0.94–1.07) ^b^	0.970		
Growth pattern (*n* = 224) ^c^	Polypoid	48.5	0.408		
	Nodular	36.2			
	Infiltrative	26.6			
Tumor location	Proximal (duodenum)	41.7	0.007 ^a^	1.34 (0.92–1.94)	0.125
	Distal (jejunum and ileum)	22.5			
Histologic type	Tubular	36.2	0.578		
	Nontubular	28.2			
Differentiation	Low grade	36.2	0.397		
	High grade	29.1			
Pancreatic invasion	Absent	36.2	0.931		
	Present	31.1			
Other loop invasion	Absent	36.2	0.044 ^a^	0.98 (0.22–4.27)	0.976
	Present	5.1			
Retroperitoneal seeding	Absent	37.4	<0.001 ^a^	2.53 (1.28–4.98)	0.007 ^a^
	Present	14.0			
Lymphovascular invasion (*n* = 231) ^c^	Absent	66.6	<0.001 ^a^	1.68 (1.33–2.50)	0.010 ^a^
	Present	17.8			
Perineural invasion (*n* = 231) ^c^	Absent	47.6	0.004 ^a^		0.329
	Present	18.7			
Margin status (*n* = 218) ^c^	No involvement	36.2	0.636		
	Involved by cancer	15.9			
Chemotherapy (*n* = 227) ^c^	Absent	37.4	0.314		
	Present	29.1			
Radiotherapy (*n* = 226) ^c^	Absent	39.7	0.005 ^a^	1.34 (0.82–2.21)	0.247
	Present	22.0			
Nodal metastasis (*n* = 215) ^c^	Absent	133.7	<0.001 ^a^		
	Present	21.6			
T category (*n* = 228) ^c^	T1-T2	– ^d^	<0.001 ^a^	1.30 (0.57–2.96)	0.528
	T3-T4	26.3			
N category (*n* = 215) ^c^	N0	133.7	<0.001 ^a^		0.001 ^a^
	N1	28.2		1.68 (1.07–2.63)	0.024 ^a^
	N2	17.8		2.41 (1.51–3.83)	<0.001 ^a^
Stage grouping (*n* = 211) ^c^	Stage I	– ^d^	<0.001 ^a^		
	Stage II	60.4			
	Stage III	21.6			
*KRAS* (*n* = 186) ^c^	Absent	39.7	0.098		
	Present	21.0			
*BRAF* (*n* = 176) ^c^	Absent	30.0	0.682		
	Present	22.6			

Hazard ratio (HR); confidence interval (CI); ^a^ significant at *p* < 0.05.; ^b^ displayed as HR with 95% CI; ^c^ only calculated using cases with available information.; ^d^ could not be calculated because >50% of the patients were alive.

## Data Availability

The data presented in this study are available on request from the corresponding author. The data are not publicly available due to the restriction of the Institutional Review Board of Incheon St. Mary’s Hospital. All other relevant data supporting study findings are within the manuscript.

## Supplementary Materials

The following supporting information can be downloaded at: https://www.mdpi.com/article/10.3390/cancers15205032/s1, Table S1: Previous studies of cyclin D1 expression in SIACs and CRCs.
