# Supplementary material for: Prognostic Significance of Cyclin D1 Expression in Small Intestinal Adenocarcinoma
Source: Cancers (Basel). 2023 Oct 18;15(20):5032. doi: 10.3390/cancers15205032 (PMC10604933; doi:10.3390/cancers15205032)
Supplement: Supplementary file 1 [file cancers-15-05032-s001.zip › cancers-2594941-supplementary.pdf]

**Supplementary Table S1.** Previous studies on cyclin D1 expression in SIACs and CRCs.

| Study | Country (yr) | IHC | *N* | Clone used | Cyclin D1$^{High}$ | | Outcome |
|---|---|---|---|---|---|---|---|
| | | | | | Cut-off level | *N* (%) | Survival |
| **SIACs** | | | | | | | |
| Present | Korea | TMA | 232 | SP4 | Score >3.5 | 145 (62.5) | Better OS |
| Aber [22] | USA (1999) | Whole section | 24 | Non-SP4[a] | ≥ 10% | 24 (30.0) | Decreased 3YSR |
| **CRCs** | | | | | | | |
| Jun [18] | Korea (2022) | TMA | 495 | SP4 | Score ≥ 1 | 389 (78.6) | Better OS and RFS |
| Jang [28] | Korea (2012) | TMA | 217 | SP4 | ≥ 30% | 158 (72.5) | Better OS and RFS |
| Ogino [29] | USA (2009) | TMA | 602 | SP4 | > 50% | 330 (54.8) | Better CSS |
| Seicean [30] | Romania (2011) | TMA | 87 | SP4 | > 10% | 47 (54.0) | Not significant |
| Stor [31] | Slovenia (2008) | TMA | 191 | SP4 | NS | NS | Not significant |
| Hilska [32] | Finland (2005) | TMA | 363 | P2D11F11 | ≥ 1% | 99 (27.3) | Better OS |
| McKay [33] | UK (2002) | Whole section | 249 | P2D11F11 | > 5% | 137 (55.0) | Better OS |
| Von Stockmar-Von Wangenheim [34] | Germany (2008) | Whole section | 200 | P2D11F11 | > 5% | 150 (75.0) | Not significant |
| Lyall [35] | UK (2006) | Whole section | 90 | P2D11F11 | > 5% | 46 (51.1) | Not significant |
| McKay [36] | UK (2000) | Whole section | 100 | P2D11F11 | > 50% | 8 (8.0) | Worse OS |
| Bhatavdekar [37] | India (2001) | Whole section | 98 | P2D11F11 | ≥ 1% | 30 (30.6) | Worse OS |
| Bahnassy [38] | Egypt (2004) | Whole section | 60 | DCS-6 | Index ≥ 6.1 | 41 (68.3) | Worse OS |
| Moore [39] | USA (2004) | Whole section | 40 | DCS-6 | > 10% | 6 (15.0) | Not significant |
| Holland [40] | UK (2001) | Whole section | 126 | DCS-6 | > 10% | 74 (58.7) | Better OS |
| Maeda [41] | Japan (1998) | Whole section | 123 | Non-SP4[a] | > 50% | 20 (16.3) | Worse OS and DFS |
| Maeda [42] | Japan (1997) | Whole section | 101 | Non-SP4[a] | > 50% | 14 (13.9) | Worse DFS |
| Tsai [43] | Taiwan (2013) | Whole section | 100 | NS | Score ≥ 2 | 49 (49.0) | Not significant |
| Belt [44] | Netherlands (2012) | TMA | 379 | NS | Score ≥ 8 | 211 (55.7) | Not significant |
| Mao [45] | China (2011) | Whole section | 169 | NS | 5% | 95 (56.2) | Worse OS |
| Fang [46] | China (2009) | TMA | 532 | NS | ≥ 10% | 380 (71.4) | Not significant |
| Kouraklis [47] | Greece (2006) | Whole section | 111 | NS | > 5% | 71 (63.9) | Not significant |
| Bondi [48] | Norway (2005) | Whole section | 219 | NS | ≥ 5% | 24 (11.0) | Not significant |
| Bondi [49] | Norway (2004) | Whole section | 162 | NS | > 5% | NS | Not significant |

Small intestinal adenocarcinoma (SIAC); colorectal carcinoma (CRC); high expression of cyclin D1 (cyclin D1$^{High}$); immunohistochemistry (IHC); tissue microarray (TMA); overall survival (OS); year-survival rate (YSR); recurrence-free survival (RFS); cancer specific survival (CSS); not stated (NS); disease-free survival (DFS).; [a]Among reports that did not precisely define the clones, three studies before novel SP4 was developed in 2004 were considered to use non-SP4.
